# Evaluating the Impact of Naltrexone on the Rat Gambling Task to Test Its Predictive Validity for Gambling Disorder

**DOI:** 10.1371/journal.pone.0155604

**Published:** 2016-05-18

**Authors:** Patricia Di Ciano, Bernard Le Foll

**Affiliations:** 1 Translational Addiction Research Laboratory, CAMH, Toronto, Canada; 2 Department of Pharmacology and Toxicology, University of Toronto, Toronto, Canada; 3 Department of Psychiatry, University of Toronto, Toronto, Canada; 4 Campbell Family Mental Health Research Institute, CAMH, Toronto, Canada; 5 Ambulatory Care and Structured Treatment Program, CAMH, Toronto, Canada; 6 Institute of Medical Sciences, University of Toronto, Toronto, Canada; 7 Department of Family and Community Medicine, University of Toronto, Toronto, Canada; VU University Medical Center, NETHERLANDS

## Abstract

Gambling Disorder has serious consequences and no medications are currently approved for the treatment of this disorder. One factor that may make medication development difficult is the lack of animal models of gambling that would allow for the pre-clinical screening of efficacy. Despite this, there is evidence from clinical trials that opiate antagonists, in particular naltrexone, may be useful in treating gambling disorder. To-date, the effects of naltrexone on pre-clinical models of gambling have not been evaluated. The purpose of the present study was to evaluate the effects of naltrexone in an animal model of gambling, the rat gambling task (rGT), to determine whether this model has some predictive validity. The rGT is a model in which rats are given a choice of making either a response that produces a large reward or a small reward. The larger the reward, the greater the punishment, and thus this task requires that the animal inhibit the ‘tempting’ choice, as the smaller reward option produces overall the most number of rewards per session. People with gambling disorder chose the tempting option more, thus the rGT may provide a model of problem gambling. It was found that naltrexone improved performance on this task in a subset of animals that chose the ‘tempting’, disadvantageous choice, more at baseline. Thus, the results of this study suggest that the rGT should be further investigated as a pre-clinical model of gambling disorder and that further investigation into whether opioid antagonists are effective in treating Gambling Disorder may be warranted.

## Introduction

Gambling Disorder is a serious health concern for those afflicted, and this is underscored by the reclassification of gambling as an addictive disorder in the recently released DSM-5. To-date, there are no approved pharmacological treatments for pathological gambling. Part of the reason why this is the case is that gambling remains difficult to evaluate in the pre-clinical stages of testing, when its early efficacy is evaluated in animal models. A number of animal models of gambling have been developed [1], but their predictive validity remains to be determined, thus making the testing of potential new treatments difficult.

The rat gambling task (rGT) [2] is a relatively novel model of rodent gambling that is based on the Iowa gambling task in humans [3]. In this task, humans are given a choice of decks of cards. Each deck of cards is associated with different probabilities of reward and punishment such that the deck with higher reward also produces higher punishment. Although the decks with the higher rewards are the ‘tempting’ options, these choices must be inhibited, as the lower rewarded decks produce the greatest number of rewards in a given time period. In rats, this is modeled in the rGT by training the rats that 4 different response holes are associated with different amounts of reward and punishment.

The purpose of the present study is to evaluate the predictive validity of the rGT by determining whether treatments that have shown some efficacy in human clinical trials also show an effect on the rGT. We decided to investigate the opioid antagonist, naltrexone, as it has been consistently shown in humans that treatment with naltrexone diminished gambling urges and behaviour in both clinical trials [4–9] and in case studies [10, 11]. These findings were supported by the further findings that nalmefene, an opiate antagonist, also reduced subjective indices of gambling in humans [12, 13]. Thus, the opiate antagonists are a useful class of drug to investigate and naltrexone was used in the present study.

In humans, pathological gamblers peforming the Iowa Gambling Task make suboptimal choices by choosing the decks with higher rewards [14], despite the overall fewer rewards obtained over a session with this option. Thus, by analogy, we have subdivided the present group of rats into two subsets, consisting of whether they preferred to make Advantageous or Disadvantageous choices at baseline [15]. It was hypothesized that naltrexone will increase Advantageous choice in the suboptimal group making more Disadvantageous choices at baseline, and have no effect on the optimal group.

## Methods

### Subjects

Subjects were 24 male Long Evans rats (Charles River, St. Constant, QC) weighing 250–300g at the start of the study. The sample size was determined based on previous studies. All animals were single-housed in a climate-controlled environment on a 12-h reverse light/dark cycle (lights off 8.00am–8.00pm) so that behavioral testing occurred during the active phase of the animals’ circadian rhythm. During behavioral testing, rats were maintained on 18–20g of rat chow per day, given after their experimental session. This results in sufficient motivation for food to ensure an adequate number of trials/sessions but also allows for continued growth during the study. Water was available *ad libitum* in their home cages. This study was carried out in strict accordance with the recommendations by the Canadian Council on Animal Care. The protocol was approved by the Centre for Addiction and Mental Health Animal Care Committee. All efforts were made to minimize suffering.

### Behavioral Equipment

Behavioral testing occurred in five-hole operant chambers (Med Associates, Roanoke, VA; the same chambers used for the five-choice serial reaction time task). The boxes were controlled by software written in Med PC running on an IBM compatible computer. The defining feature of such boxes is that an array of five stimulus–response holes is located on one wall of the chamber, although only the four outer holes are used during the task. Each response hole can be illuminated by a stimulus light located therein, and nose-poke responses into a hole are detected by an infrared sensor. A food tray, also equipped with an infrared sensor and a tray light, is located in the middle of the opposite wall, into which pellets can be delivered via an external pellet dispenser. The entire chamber can also be illuminated using a house-light.

### The Rat Gambling Task

In the rGT, animals learn about which response option has which size of reward and probability/duration of a time-out punishment (BioServ 45 mg Rodent Purified Diet, product #F0021). For details of the training, see [2]. Briefly, rats were first trained on the 5 choice serial reaction time task (5-CSRT) for 13 days, followed by 7 days of forced choice training. Forced choice training was essentially the same as the 5-CSRT task, but each hole was associated with a different probability of rewards and punishment (see Table 1). The purpose of forced choice training was for the rat to learn which hole produced which size and probability of reward and punishment. After forced choice, rats began training on the rGT.

**Table 1:** Reward and punishment received for the various response options in the rGT.

**Group A**	hole 1	hole 4	hole 5	hole 2
**Group B**	hole 2	hole 5	hole 4	hole 1
**Choice**	P1	P2	P3	P4
**Reward (# pellets)**	1	2	3	4
**Punishment Duration**	5s	10s	30s	40s
**Punishment Probability**	0.1	0.2	0.5	0.6
**Rewards Possible**	295	411	135	99

In the rGT, animals initiate each trial by making a nosepoke response at the food tray (with a traylight). This triggers the start of a 5 s inter-trial interval (ITI) before the stimulus lights are turned on in all of the four active holes. A response at one of the illuminated holes results in the offset of all the stimulus lights and either delivery of the pre-set amount of reward for that hole option, or the start of the time-out “punishment” period. Sessions lasted for 30 minutes. The following is a summary of possible outcomes:

1. Reward: If the animal is rewarded on any trial, food delivery is signalled by onset of the traylight which remains illuminated until the animal collects the reward. Responding at the food tray also initiates the start of the next trial. The size of reward and punishment probability/duration is given in Table 1.
2. Punishment: If the animal is punished, the traylight remains off until the end of the time-out period, whereupon this light is turned on to signal that the animal can initiate the next trial. During these time-out periods, the stimulus light within the hole chosen on that trial flashes at a frequency of 0.5 Hz. The size of reward and punishment probability/duration is given in Table 1.
3. Omission: If the animal fails to make a response within 10 seconds after the four stimulus lights are turned on, the stimulus lights are turned off, the trial is scored as an omission and the traylight is illuminated to signal the beginning of the next trial. Animals are not punished for omitting trials.
4. Premature responses: Premature responses made at the array during the ITI are punished by a 5 s time-out period during which no further trials can be initiated. The duration of the time-out is signalled by illumination of the houselight, and terminated by onset of the traylight so that animals can begin another trial.

Training continued until a stable pattern of choice between the four options was observed (approximately 30 sessions). Animals were randomly assigned to one of two groups (A or B), receiving a different configuration of response outcomes in the holes (Table 1).

### Drug

Naltrexone (Sigma-Aldrich, Oakville, ONT, Canada) was administered acutely at doses of 0, 1, 3, or 10 mg/kg, i.p. and given in counterbalanced order at a volume of 1ml/kg. Naltrexone was dissolved in sterile saline, aliquoted and frozen (no longer than one month) and administered i.p. 30 minutes prior to the test session on the day of testing. Doses of naltrexone were chosen based on previous studies [16]. Doses were administered in a Latin-square design. Prior to receiving any naltrexone injections, rats were given 0.3 ml of saline i.p. for at least 2 days to habituate them to the testing procedure. At least two days of stable responding on the rGT without any treatment separated each naltrexone treatment day. Rats were trained on the rGT for approximately 30 days prior to the start of drug testing.

### Data Analyses

#### Measures

The measures chosen are based on Zeeb et al. (2009) [2], which are predicated by studies of the 5 Choice Serial Reaction Time Task. For a discussion of the interpretation of these measures, see Robbins (2002) [17]. The measures collected are: 1) the percentage of trials on which an animal chose a particular option (number of choices of a particular option /number of trials (including omissions) *100). Choice data was arcsine transformed [2]; 2) The total number of trials initiated; 3) Omissions made expressed as a percentage of the total number of trials initiated; 4) Premature responses expressed as a percentage of the total number of trials initiated; 5) The latency to make a choice after initiation of a trial; 6) The latency to collect reward after making a response choice; 7) Perseverative responses on punished and rewarded trials calculated as the fraction of the total punishment duration or total number of trials rewarded

#### Choice and Optimal vs Suboptimal

In the rGT, rats must learn the optimal strategy to obtain the most number of reinforcements per session as possible. To achieve this, rats must inhibit the choice that produces large rewards because this also results in the greatest punishment; choices with smaller rewards produce more pellets per session. In the rGT, there are four choice options, P1, P2, P3 and P4 that produce 1, 2, 3 or 4 pellets, respectively. If the rats choose exclusively one option, the number of pellets possible would be greatest with P2 (411), then with P1 (295), then P3 (135), with P4 (99) producing the fewest number of pellets (see Table 1) [2]. Thus, P1 and P2 are optimal strategies, while P3 and P4 are suboptimal ‘tempting’ strategies. In the Iowa gambling task, it is known that pathological gamblers choose these ‘tempting’ options [14, 18]. Therefore, the percent choice of P1 was added to the percent choice of P2 to comprise the ‘Advantageous’ option, while P3 and P4 percent choices were summed to produce the ‘Disadvantageous’ option. Rats that made more advantageous choices (50% advantageous choice or greater) under vehicle were analyzed as the ‘Optimal’ group, while those that made more disadvantageous than advantageous choices (50% disadvantageous choices or greater) under vehicle were the ‘Suboptimal’ group.

#### Analysis of Choices P1 to P4

The effects of naltrexone on percent choice for each of P1, P2, P3 and P4 was analyzed with a Dose (4 levels) X Group (2 levels; Optimal, Suboptimal) X Choice (4 levels; P1, P2, P3, P4) mixed ANOVA with Group as the between-subjects factor. A significant interaction was followed by *t*-test with Bonferroni correction.

#### Analysis of Optimal vs Suboptimal Choices and Other Measures

For all measures, data was analyzed with two-way mixed ANOVAs on Dose (4 levels) X Group (2 levels). Significant effects were followed up with *t*-tests using the Bonferroni correction. All statistical analyses were conducted using SPSS for Windows with a criteria for significance of p<0.05.

#### Distribution of Responses as Either Optimal or Suboptimal

To determine the distribution of either optimal or suboptimal responding, the percentage of advantageous choices made was presented as a frequency distribution at either 0–10, 11–20, 21–30, 31–40, 41–50, 51–60, 71–80 or 91–100 percent of advantageous choices.

#### Stability of Responding

To demonstrate stability of responding across testing days, the percent of advantageous choices on the day prior to any drug treatments was compared to the percent of advantageous choices made on the day prior to the last drug treatment. Data was analyzed with a Group (2 levels; Optimal vs Suboptimal) X Day (2 levels; day before first drug treatment vs day before last drug treatment) mixed ANOVA with Group as the between-subjects factor.

## Results

The final group sizes were optimal = 15 and suboptimal = 9. All rats consumed all food pellets after all sessions.

### Analysis of Choice

**Fig 1:**
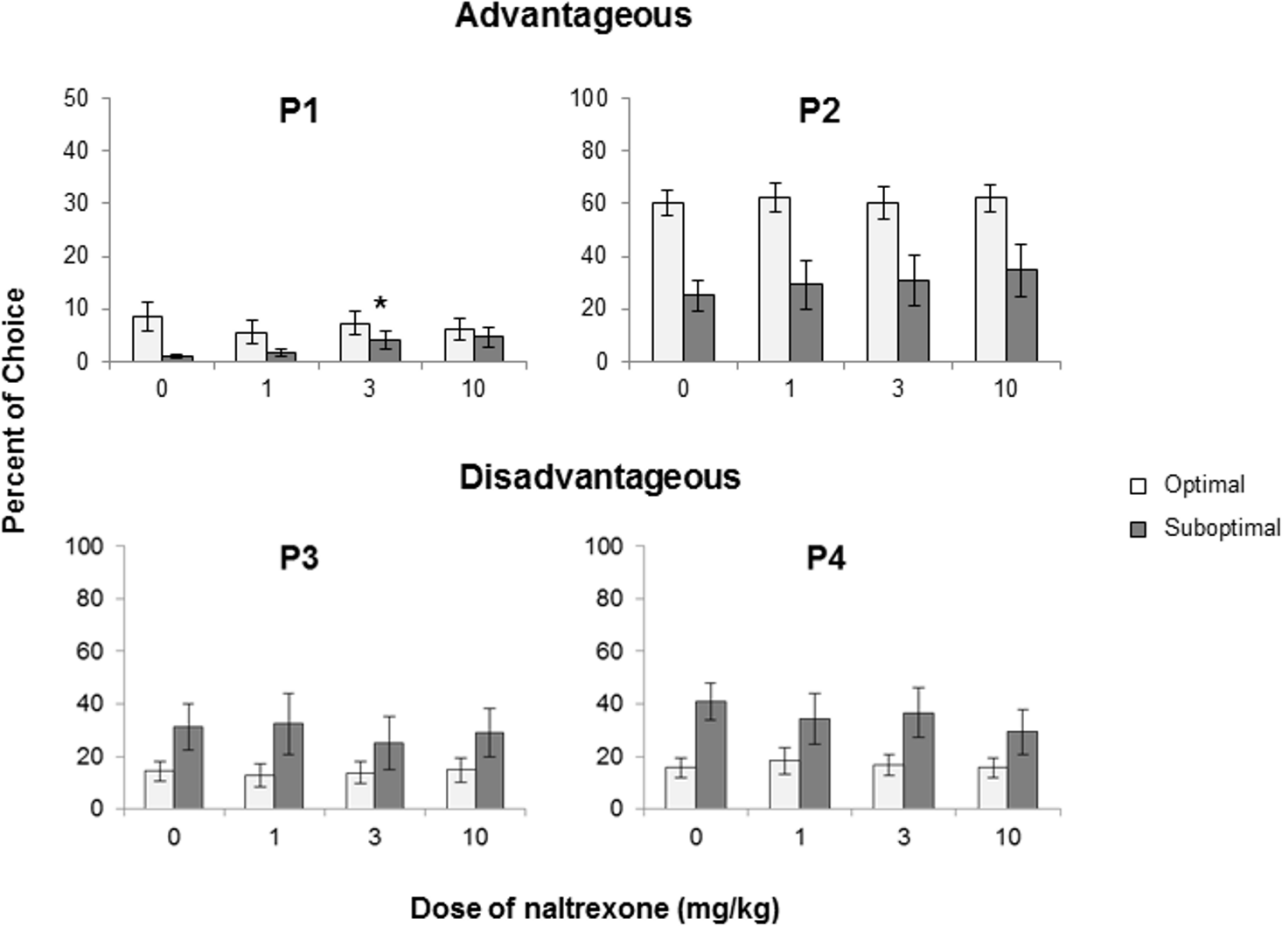
**Mean + SEM percent choice of the P1 (top left panel), P2 (top right panel), P3 (bottom left panel) and P4 (bottom right panel) options.** Data are presented for the Optimal (n = 15; open bars) and Suboptimal (dark bars, n-9) groups. Dose X Group ANOVAs revealed a significant interaction for P1 (F(3, 66) = 3.256, p_GG_ = 0.047). *significant *t*-tests after correction for multiple comparisons.

A Group X Dose X Choice ANOVA revealed an overall interaction that approached significance (F(9, 198) = 1.823, p = 0.066; Fig 1). Dose X Group ANOVAs revealed a significant interaction for P1 only (F(3, 66) = 3.256, p_GG_ = 0.047). Follow-up *t*-tests revealed that the difference between vehicle and the 3mg/kg (t(8) = -3.298, p = .011) and vehicle and the 10 mg/kg dose (t(8) = -2.328, p = 0.048) were significant (p<0.05) for the suboptimal group, indicating that naltrexone increased responding for P1 in the Suboptimal rats. Only the comparison of 3 mg/kg to vehicle was significant after correction for multiple comparison (p<0.0167). See Fig 1.

### Advantageous, Disadvantageous Responding and Other Measures

**Fig 2:**
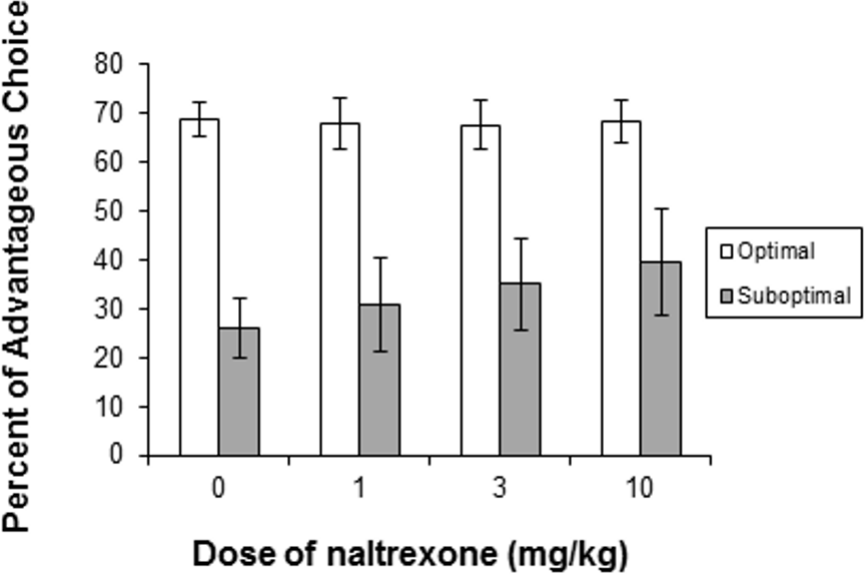
Mean + SEM percent choice for the advantageous choice at different doses of naltrexone for the Optimal (open bars, n = 15) and Suboptimal (dark bars, n = 9) groups. Group X Dose ANOVAs revealed a significant interaction for Advantageous responding (F(3, 66) = 2.988, p = 0.037).

Analysis of behavioral measures with Group X Dose ANOVAs revealed a significant interaction for Advantageous responding (F(3, 66) = 2.988, p = 0.037; Fig 2). Comparison of each dose to vehicle with *t*-tests revealed no significant effects, but the comparison of 10 mg/kg to vehicle approached significance for the disadvantageous group, when not corrected for multiple comparisons (p = 0.056). Analysis of other measures with Dose X Group ANOVAs did not reveal any significant interactions, but a main effect of Group was found for number of trials (F(1, 22) = 6.110, p = 0.022) (Table 2), indicating that the Suboptimal group initiated fewer trials than the Optimal group.

**Table 2:** Effect of naltrexone on other measures of the rGT for the Optimal (n = 15) and Suboptimal (n = 9) groups. Dose X Group ANOVAs revealed no significant interactions or main effects. Data presented are mean ± SEM. *Indicated a main effect of Group (p<0.05).

VARIABLE	GROUP	DOSES (mg/kg)			
		Vehicle	1	3	10
Trials	Optimal*	89.55 ± 6.11	93.96 ± 8.62	97.88 ± 7.85	90.1 ± 7.43
	Suboptimal	64.89 ± 3.07	66.34 ± 4.24	72.13 ± 5.80	71.9 ± 8.99
Omissions	Optimal	0.97 ± 0.35	0.81 ± 0.39	1.89 ± 1.70	1.02 ± 0.60
	Suboptimal	2.16 ± 1.14	2.12 ± 1.47	2.98 ± 1.62	2.22 ± 1.28
Premature Responding	Optimal	12.92 ± 1.91	10.52 ± 1.52	8.46 ± 1.58	10.28 ± 1.68
	Suboptimal	13.17 ± 3.42	15.87 ± 4.08	12.20 ± 3.17	19.01 ± 3.97
Reward Perseverative	Optimal	.020 ± .01	.021 ± .01	.012 ± .01	.011 ± .003
	Suboptimal	.014 ± .01	.032 ± .02	.014 ± .01	
					.010 ± .004
Punishment Perseverative	Optimal	.051 ± .01	.051 ± .01	.048 ± .01	.058 ± .01
	Suboptimal	.032 ± .01	.044 ± .01	.039 ± .01	.042 ± .01
Choice Latency	Optimal	1.38 ± .35	1.39 ± .19	1.27 ± .14	1.37 ± .20
	Suboptimal	0.93 ± .28	1.21 ± .21	1.28 ± .20	1.14 ± .22
Collect Latency	Optimal	1.76 ± .11	1.80 ± .16	1.64 ± .10	1.73 ± .13
	Suboptimal	1.64 ± .21	1.37 ± .09	1.41 ± .08	1.94 ± .65

### Distribution of Responses as Either Optimal or Suboptimal

**Fig 3:**
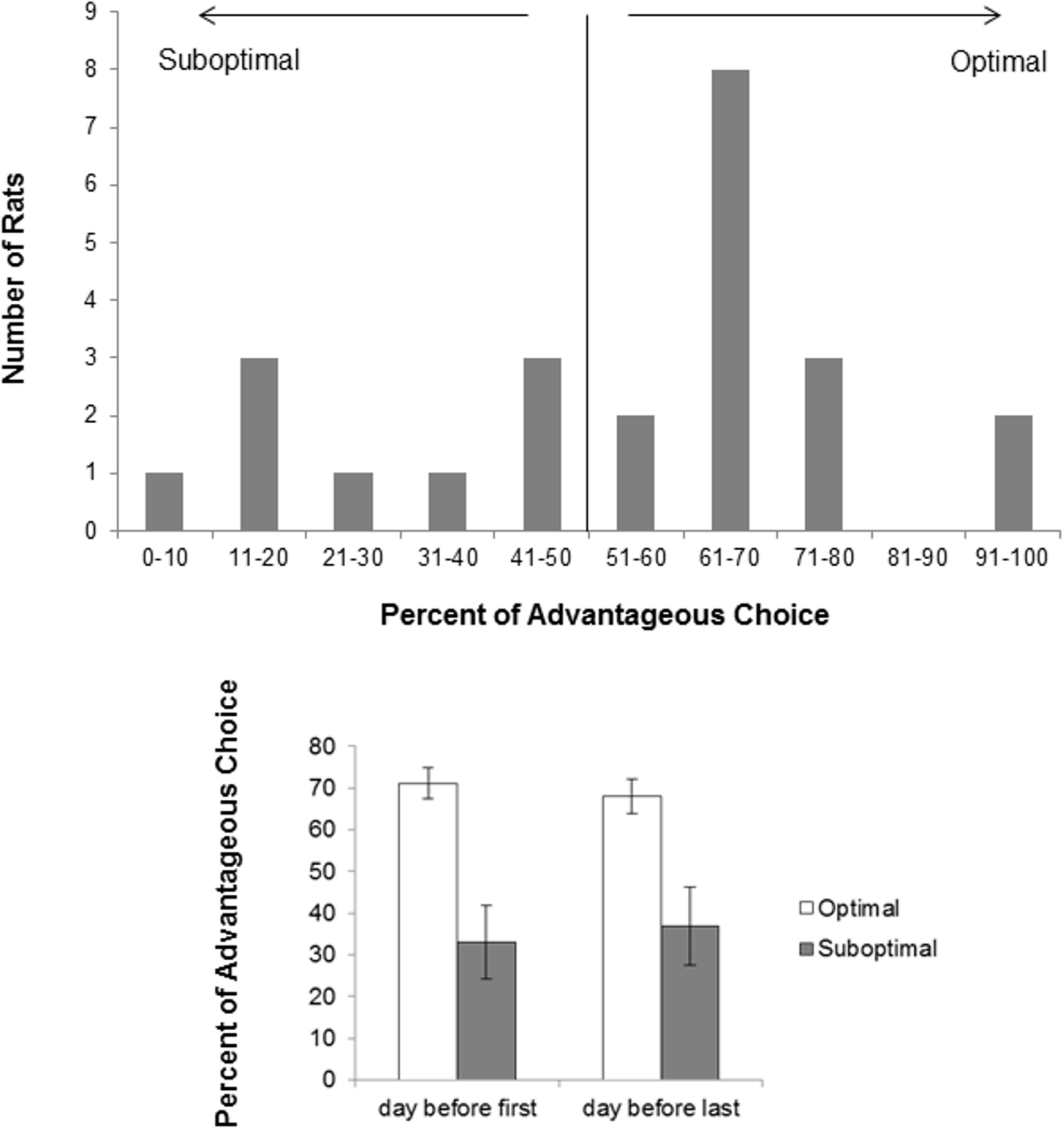
Top panel: Distribution of percent of Advantageous responding made by rats. Optimal rats were those that made more Advantageous responses during a session. Bottom panel: Mean ± SEM percent of advantageous responding on the day before the first drug treatment and the day before the last drug treatment. Data are presented for the Optimal group (open bars, n = 15) and Suboptimal group (dark bars, n = 9). Stability of responding was demonstrated by the lack of any significant effects.

To determine the distribution of either optimal or suboptimal responding, the percentage of advantageous choices made was presented as a frequency distribution (Fig 3). The percentage of advantageous responding was evenly distributed, but had a peak at 61–70% of advantageous responses. Eight rats made between 60–70% of advantageous responses, and 1–3 rats made either 0–10, 11–20, 21–30, 31–40, 41–50, 51–60, 71–80 and 91–100 percent of advantageous choices. This peak at 61–70% accounted for 8 of the 15 rats in the optimal group.

### Stability of Responding Over Test Days

Analysis of the percent Advantageous choice made either prior to the first or last day of treatment (Fig 3), revealed no significant Group X Day interaction, but a main effect of Group was found (F(1, 22) = 14.812, p = 0.001), indicating that the Optimal group made more Advantageous choices than the suboptimal group.

## Discussion

The present study found that naltrexone improved choice behavior in a subset of rats that made fewer advantageous choices at baseline. It had no effect on rats that made more advantageous choices at baseline. These effects were selective to choice behavior and did not impinge on any other measures of the rGT. Specifically, the choice P1 was most affected by naltrexone in the Suboptimal rats, with an increase in advantageous choices being seen. The responding of rats was stable across days.

The effect of naltrexone to increase advantageous choices in the suboptimal rats reflects an effect primarily on P1 responses, as significant increases were also seen in P1 responses in the suboptimal group. A lack of effect in the optimal group is not likely due to a ceiling effect, as the overall percentage of P1 responses is lower than it is for the other options, and thus, it is possible that an increase in responding in this group could have been observed if it existed. Instead, an effect on the suboptimal group suggests that some aspect of decision-making was impaired. This conclusion should be somewhat tempered by the fact that no effects were observed in *post hoc* tests of vehicle to different doses when overall responses on the disadvantageous choice was analyzed. Indeed, overall main effects were significant, despite the relatively small sample size (n = 9).

The rGT is a novel animal model of gambling that is based on the IGT [3]. In this model, animals are required to inhibit ‘tempting’ options with higher reward (and higher punishment) in favor of response options with fewer rewards that produce more food pellets over time. Some rats make more advantageous choices over sessions, while others prefer the disadvantageous options. Analysis of the percentage of responses of either advantageous or disadvantageous responses made revealed that responding over days remained stable from the start of the dose-response to the end of the dose-response. The distribution of rats into ‘Optimal’ or ‘Suboptimal’ groups was evenly distributed over choice options, with the most number of rats making between 60–70% of advantageous responses in a given session. Analysis of differences between the Optimal and Suboptimal groups revealed that differences were found only on the Advantageous/Disadvantageous choice and number of trials. The fewer number of trials initiated by the Suboptimal group likely reflects the greater time-out imposed on the choices with higher reward/punishment outcomes. A lack of effect on other measures suggests that these rats did not vary on impulsivity or food motivation. Instead, if these rats represent different subpopulations, differences may be due to different response strategies. Future investigations will need to further explore this.

Future investigations should further delineate the effect of opiate antagonists on models of gambling behavior, as the present study provides some evidence that naltrexone may be effective in problem gamblers, with no impact on those who adopt healthy gaming. This is an important consideration given that many treatments for substance use disorders have had the problem of side effects that make compliance a challenge [19]. Indeed, no other measures apart from choice were affected in the present study. To the extent that choice in this task represents the same cognitive impairment in the Iowa gambling task [3], this model provides face and construct validity, and the present findings are encouraging in terms of the predictive validity of this task.

The present findings support a previous study in which no effect of naltrexone was seen on a delayed discounting task [20]. The delay discounting task is a measure of impulsivity and this suggests that naltrexone does not influence impulsivity, consistent with the present finding of no effect on premature responding. Similarly, it is not likely that any effects of naltrexone were due to its ability to affect the palatability of food [21], as responding was increased, while opiate antagonists have primarily been found to decrease, food intake in animal models [22, 23]. Similarly, any effects of naltrexone are not likely due to changes in motor activation, as naloxone has been found to decrease [24], or have no effect [25], on motor activation, effects that are opposite in direction to the present findings. Naltrexone has been found to have some effects on memory [26–28] and to improve performance on the Morris water maze in rats with vascular dementia [29]. The latter finding is compelling in light of the present findings as it suggests that naltrexone may increase cognitive performance in populations with impairments. Future studies will need to investigate the effects of opiate antagonists on individual differences in performance.

The present findings are interesting in view of a literature demonstrating that naltrexone decreases measures of seeking and taking of drugs of abuse. For example, naltrexone decreased ethanol drinking in acute [16, 30–32] and chronic [33] models of ethanol consumption, (but see [34, 35]) and also lowered intake of heroin or cocaine/heroin combinations in a self-administration model [36]. Context-induced [37] and ethanol-induced [32] reinstatement of ethanol-seeking as well as amphetamine-induced reinstatement [38] and sensitization [39] were also attenuated. Further findings that naltrexone did not affect food and water intake [16, 31, 38], or basal locomotion [39] suggest that effects were on some aspect of the addictive properties of the drugs and may provide an account of the present data in terms a cognitive impairment rather than some aspect of satiety or food reward, *per se*.

In humans, few pharmacological treatment approaches have been used successfully in clinical trials for the treatment of gambling with the exception perhaps of opiate antagonists such as naltrexone [40, 41]. That is, patients treated with naltrexone [4, 6] or another opiate antagonist, nalmefene [12, 13], showed improvement in measures of gambling. Although other medications have shown some efficacy, it was concluded in a meta-analysis that the effects of antidepressants and mood stabilizers may vary based on comorbidity [42]. Indeed, in healthy people, naltrexone was better than topiramate, bupropion or escitlaopram [7]. Acamprosate and baclofen [43] were not effective, while topiramate, bupropion and fluvoxamine had mixed results [8, 9, 44–48]. It should be noted that, in all these studies, participants met the DSM diagnosis for gambling disorder. It is not known whether naltrexone would affect people with healthy gaming, but based on the present results, it can be hypothesized that naltrexone would not have an effect on this population.

The one other drug that has shown some promise for the treatment of gambling disorder is disulfiram. Traditionally used as a medication for the treatment of alcohol use disorder through inhibition of alcohol dehydrogenase, disulfiram has recently been shown to be effective in cocaine use [49–54] suggesting it may have an additional mechanism of action. Based on this, it has been proposed that disulfiram may be an effective treatment for gambling disorder [55]. Indeed, disulfiram has shown promise in reducing gambling in one case report [56]. In another study, no decreases in gambling were observed, but cravings were decreased [57]. Further studies should explore the impact of disulfiram on the rGT.

## Conclusions

In conclusion, the present study provides some support for the contention that opiate antagonists may serve as treatments for gambling disorder. It further suggests that the rGT may be used as a pre-clinical method for screening of new drugs. Although compelling as an animal model, the present study is underpowered to do a complete analysis as to the validity of the model and the use of optimal and suboptimal subgroups. In our studies, we have observed that different cohorts of rats have different numbers of optimal and suboptimal rats and that the group differences on measures can vary when the sample sizes are relatively small, as they are in this study for the suboptimal group. Thus, future studies will need to further evaluate the utility of this model for the pre-clinical evaluation of drugs that are in development for the treatment of gambling disorder. Further evaluation of naltrexone as a treatment option for Gambling Disorder is warranted.
